# Computer Vision Syndrome among Patients Attending the Outpatient Department of Ophthalmology in a Tertiary Care Centre: A Descriptive Cross-sectional Study

**DOI:** 10.31729/jnma.5123

**Published:** 2020-10-31

**Authors:** Priyanka Shrestha, Pranil Man Singh Pradhan, Om Krishna Malla

**Affiliations:** 1 Department of Ophthalmology, Kathmandu Medical College Teaching Hospital, Kathmandu, Nepal; 2 Department of Community Medicine, Institute of Medicine, Tribhuvan University, Kathmandu, Nepal

**Keywords:** *computer*, *vision syndrome*, *ergonomics*

## Abstract

**Introduction:**

Computers and digital electronic devices have become an integral part of life. These devices have adverse effects and nowadays are considered leading occupational hazards. Computer vision syndrome comprises of all the ocular, visual and musculoskeletal symptoms secondary to long term computer use. The objective of this study is to determine the prevalence of computer vision syndrome among people attending the outpatient department of ophthalmology in the tertiary care center in Nepal.

**Methods:**

A descriptive cross-sectional study was done among 70 patients in a tertiary care hospital from January 2017 to June 2017 after obtaining ethical approval from the institutional review committee (Ref: 12042017). Convenient sampling method was applied and the point estimate at 95 % confidence interval was calculated along with frequency and proportion for binary data. Patients using computers for more than one hour were included in the study. All data were entered in Microsoft Excel and analyzed using statistical package for social sciences version 20.

**Results:**

Among 70 patients, 67 (95.7%) (87.9-99.1 at 95% confidence interval) had one or more symptoms on computer use. The mean duration of computer use was 7.5±5.4 years and average hours of computer use among computer users were 6.9±3 hours. The most common symptom among computer users was headache seen in 46 (62.2%) patients.

**Conclusions:**

Our study showed that a significant number of people using a computer develop one or more symptoms on the long-term use of the computer. Therefore, it is very important to create awareness regarding computer vision syndrome and methods to prevent it among computer users.

## INTRODUCTION

Computers and digital electronic devices are being largely used nowadays not only in the form of the desktop at the workplace but also in the form of laptops, tablets, and smartphones. Both adults and children use it for email, entertainment, communication, work, gaming, etc.^1^ Their use are not without adverse effects. Computer Vision Syndrome (CVS) is a rapidly growing problem among Video Display Terminal (VDT) users among both professional and ordinary computer users.

American Optometric Association in 1995 defined CVS as “the complex of eye and vision problems related to near work which are experienced during or related to computer use.”^2^ The ocular complaints include eye strain, eye fatigue, irritation, redness, dry eyes, blurred vision, and burning sensation and non-ocular symptoms include headaches, shoulder, neck and back pain.^3^ Therefore, the objective of this study is to determine the prevalence of computer vision syndrome among people attending the outpatient department of ophthalmology in the tertiary care center in Nepal.

## METHODS

A descriptive cross-sectional study was done among 70 patients in Kathmandu Medical College from January 2017 to June 2017 after obtaining ethical approval from the institutional review committee of Kathmandu Medical College Teaching Hospital (Ref: 12042017). The convenient sampling method was applied. The inclusion criteria were patients aged 18 to 39 years who visited the Ophthalmology outpatient department of Kathmandu Medical College Teaching Hospital. The patients who did not give consent were excluded from the study.

Sample size calculation,

$$\begin{array}{ll} \text{n} & \text{= }{\text{Z}}^{\text{2}}\times \text{p}\times \text{(1}-\text{p)}/{\text{e}}^{\text{2}}\\ & \text{= }{\left(1.96\right)}^{\text{2}}\times 0.79\times 0.21/{\left(0.01\right)}^{\text{2}}\\ & \text{= }0.64/0.01\\ & =64 \end{array}$$

Where,

n = sample size
Z = 1.96 at 95% confidence interval (CI)
p = prevalence, 79%^4^
e = margin of error, 10%

Therefore, the sample size calculated was 64. Taking a non-response rate of 10%, the total sample size was 70.

All subjects were requested to fill a written questionnaire regarding their computer use and symptoms observed during computer use. The questionnaire included age, sex, average working hours a day, type of work, total time (in months/years) of work on the computer, history of eye problems and/or consultation with an eye doctor, use of computer eyeglasses, refractive correction (glasses), use of an antiglare screen, brightness adjustment, windows position, idea of CVS, symptoms experienced, non-ocular problems (muscle pain) and relief measures. A complete ophthalmological examination was done which included best-corrected visual acuity, refractive error, anterior and posterior segment evaluation, dry eye evaluation, convergence, accommodation, and fundus examination. The questionnaire, used in similar previous studies, was tailored for our study.^5^

All data were entered in Microsoft Excel and analyzed using Statistical Package for Social Sciences (SPSS) Version 20. Point estimate at 95% confidence interval was calculated. Descriptive statistics, frequency tables and percentages were used.

## RESULTS

A total of 70 computer users were enrolled in the study. Among the computer user, 67 (95.7%) (87.9-99.1 at 95% confidence interval) had one or more symptoms on computer use. The mean age was 25.64±5.6 years. Among them 41 (58.6%) were male and 29 (41.4 %) were female. Most of the study participants 43 (60.8%) were within the age group 20-29 years. Refractive error was seen in 36 (51.4%) among which 20 (28.6%) were diagnosed with refractive error for the first time (Table 1).

**Table 1:** Distribution of refractive error among computer users.

Types of refractive error	n (%)
Simple Myopia	23 (35.3)
Simple Hypermetropia	2 (3)
Simple Myopic Astigmatism	20 (30.7)
Simple Hyperopic Astigmatism	6 (9.2)
Compound Myopic Astigmatism	12 (18.4)
Mixed Astigmatism	2 (3)

*n = the total number of eyes with refractive error

The mean duration of computer use was 7.5±5.4 years (Table 2).

**Table 2:** Distribution of duration of computer use.

Duration (in years)	n (%)
1-5	30 (42.8)
6-10	25 (35.7)
11-15	8 (11.4)
16-20	5 (7.1)
21-25	2 (2.8)

The average hours of computer use among computer users were 6.9±3 hours (Table 3).

**Table 3:** Distribution of hours of use of computers among computer users.

Hours of computer use	n (%)
1-5	22 (31.4%)
6-10	43 (61.4%)
11-15	4 (5.7%)
16-20	1 (1.4%)

Among the computer users, 40 (57.1%) used laptops only 9 (12.9 %) used desktop only and 21 (28.4%) used both laptops and desktops. Most participants used electronic devices for internet use 52 (74.3%) followed by emails and programming in 43 (61.4%) and 23 (32.9%) respectively. The mean duration of computer use after which the subjects developed symptoms was 3.2 hours. The most common symptom among computer users was headache seen in 46 (62.2%) (Table 4).

**Table 4:** Distribution of various symptoms after computer use among computer users.

^*^Symptoms	n (%)
Headache	46 (62.2)
Dry Eye	36 (51.4)
Neck pain /shoulder pain /back pain	36 (51.4)
Redness	32 (45.7)
Blurring of vision	40 (57.1)
Burning sensation	31 (41.9)

* Multiple responses

The most common preventive measures taken by the computer user was taking frequent breaks in 57 (64%) (Table 5).

**Table 5:** Distribution of various preventive measures taken by computer users.

^*^Preventive measures	n (%)
Taking frequent breaks	57 (64)
Use of eye drops	14 (15.7)
Use of screen filters	5 (5.6)
Looking at far objects	3 (14.6)
No measures are taken	6 (6.3)

* Multiple responses

Among the computer users, the Schirmers test done after the application of topical anesthesia showed a dry eye in 18 (25.7%). Tear film break-up time done in all patients showed values less than 10 in 23 (32.9%). Convergence insufficiency was found in 6 (8.6%) of subjects with computer vision syndrome which was treated accordingly. Among the computer users, 17 (24.3%) placed the computer screen at a distance of 20-25 inches and 19 (27.1%) had their screen slightly below the eye level.

## DISCUSSION

Computer and visual display terminal have now been a part of our lives. It is said to be the leading cause of occupational hazards of the 21^st^ century.^6^ Among the 70 computer users, 95.7 % had one or more symptoms following the use of a computer continuously for more than one hour. Various studies have shown the prevalence of computer vision syndrome ranging from 46.3%-89.9% among the various group of subjects using computers.^4,5,7,8^ The higher prevalence of computer vision syndrome in our study could be due to the inclusion of back, neck, and shoulder pain in the diagnosis of computer vision syndrome, and also we have not taken any specific duration of symptoms for the diagnosis of computer vision syndrome.

The mean age of presentation of our participants was 25. Sixty-four patients between 20 to 30 years of age, the observation of which was on par with the mean age presented in other studies done among different groups of participants.^9–11^ The most common symptom among computer users was headache (62.2%) followed by dry eye and neck, shoulder (51.4%), and back pain (51.4%). Headache is a common symptom among computer users in various studies the prevalence ranging from 43%-82.1%.^4,8^

Working at a computer screen requires greater visual effect than reading a paper which is due to a lesser blink rate, about 7 blinks per minute as compared to a normal blink rate of 22 per minute.^6^ Therefore, longer screen time leads to dry eye and discomfort on computer use. Schirmer's test showed a dry eye in 25.7% and lower tear film break-up time in 32.9% which may indicate that evaporative dry eye is more common in subjects with computer vision syndrome. Lower tear film break up time in long term computer users was statistically significant than in control in a study done by Akkaya et al.^12^ Among the computer users 75.7% were aware of the adverse effects of long term computer use and 64% among them had been taking frequent breaks while using the computer the results of are similar to a study by Akinbinu et al.^13^

Refractive error was seen in 51.4% of the computer users and spherical error was present in 38.3% and astigmatism was present in 61.3% the results of which did not match with a study done in Nigeria which studied the refractive error among computer users, showed more of spherical error than astigmatic error among the computer users.^14^ Among the subjects with refractive error, 28.6% had not been corrected for their error which could be a reason for their symptoms while using computers. Since our subjects had more of an astigmatic error and many of them were not corrected for their refractive error, could be one reason a large number of them experiencing symptoms during computer use and a major proportion of them experiencing asthenopic symptoms most commonly headache.

Various ergonomic recommendations have been made to prevent computer vision syndrome which recommends at least a one-arm distance between the computer screen and eye (20-25 inches), the position of the gaze of an eye to the screen being slightly downward, and the use of antiglare screen filters.^15^ However only 24.3% placed the computer screen at a recommended distance, 27.1% had their screen placed slightly below the eye level and only 5.6% used anti-glare screen filters. This shows that the subjects in our study did not follow the recommended ocular ergonomics which could be a cause of a higher number of them suffering from computer vision syndrome.

The limitations of our study were small sample size and since the design of the study was a cross-sectional study causal attribution could not be made. Therefore, generalization to the population cannot be done.

## CONCLUSIONS

The prevalence of computer vision syndrome among computer users in our study was 95.7%. Refractive error was seen in 51.4% and the most common symptom was a headache. The presence of a higher rate of refractive error with many being diagnosed for the first time, the inclusion of musculoskeletal symptoms for the diagnosis of computer vision syndrome, and poor adherence to computer ergonomics could be associated with a higher rate of computer vision syndrome in our study.
